# STLV-1 co-infection is correlated with an increased SFV proviral load in the peripheral blood of SFV/STLV-1 naturally infected non-human primates

**DOI:** 10.1371/journal.pntd.0006812

**Published:** 2018-10-01

**Authors:** Sandrine Alais, Amandine Pasquier, Brice Jegado, Chloé Journo, Réjane Rua, Antoine Gessain, Joelle Tobaly-Tapiero, Romain Lacoste, Jocelyn Turpin, Renaud Mahieux

**Affiliations:** 1 International Center for Research in Infectiology, Retroviral Oncogenesis laboratory, INSERM U1111 –Université Claude CNRS, UMR5308, Ecole Normale Supérieure de Lyon, Université Lyon, Lyon, France; 2 Equipe labellisée “Ligue Nationale Contre le Cancer” et Labex Ecofect, Lyon, France; 3 Ecole Pratique des Hautes Etudes, Paris, France; 4 Epidémiologie et Physiopathologie des Virus Oncogènes, CNRS UMR 3569, Pasteur Institute, Paris, France; 5 INSERM U944-CNRS Université Paris Diderot, UMR7212-IUH-Hôpital St-Louis, Dynamic of Retroviruses and Retrotransposons Group, Paris, France; 6 Station de Primatologie-UPS846-CNRS, France; 7 Section of Virology, Department of Medicine, Imperial College London, London United Kingdom; Institute for Disease Modeling, UNITED STATES

## Abstract

Simian T-Leukemia Virus type 1 and Simian Foamy Virus infect non-human primates. While STLV-1, as HTLV-1, causes Adult T-cell Leukemia/lymphoma, SFV infection is asymptomatic. Both retroviruses can be transmitted from NHPs to humans through bites that allow contact between infected saliva and recipient blood. Because both viruses infect CD4+ T-cells, they might interfere with each other replication, and this might impact viral transmission. Impact of STLV-1 co-infection on SFV replication was analyzed in 18 SFV-positive/STLV-1-negative and 18 naturally SFV/STLV-1 co-infected *Papio anubis*. Even if 9 animals were found STLV-1-positive in saliva, STLV-1 PVL was much higher in the blood. SFV proviruses were detected in the saliva of all animals. Interestingly, SFV proviral load was much higher in the blood of STLV-1/SFV co-infected animals, compared to STLV-1-negative animals. Given that soluble Tax protein can enter uninfected cells, we tested its effect on foamy virus promoter and we show that Tax protein can transactivate the foamy LTR. This demonstrates that true STLV-1 co-infection or Tax only has an impact on SFV replication and may influence the ability of the virus to be zoonotically transmitted as well as its ability to promote hematological abnormalities.

## Author summary

Foamy viruses infect a lot of mammalian hosts including non-human primates (NHP) and humans. Foamy infection is not associated with disease, although a recent report described hematological abnormalities in infected humans. Some NHP species are also naturally infected with another retrovirus i.e. Simian T lymphotropic virus type 1, while humans are infected with the Human T lymphotropic virus type 1 counterpart. Both viruses cause leukemia. Here we report that natural foamy/STLV-1 co-infection is associated with a higher foamy virus proviral load in blood. Co-infected animals might therefore present a higher risk of developing hematological disease.

## Introduction

Human T-cell Leukemia Virus Type 1 (HTLV-1) infects 5 to 20 million individuals worldwide [1]. This retrovirus promotes CD4+ T-cell proliferation and causes either a lymphoproliferative disease named Adult T-cell Leukemia/lymphoma (ATL) [2, 3] or a neurological disease called HTLV-1-Associated Myelopathy/Tropical Spastic Paraparesis [4, 5]. Simian T-Lymphotropic Virus type 1 (STLV-1) is the simian counterpart of HTLV-1. It is transmitted among non-human primates (NHPs) through sexual intercourses as well as through bites [6, 7]. It is also transmitted to humans after bites [8]. STLV-1 and HTLV-1 strains from the same regions are usually almost undistinguishable at the nucleotide level and more than thirty Old World NHP species, including baboons, are infected with STLV-1 [9–13]. Even if STLV-1 has been infecting NHPs for millennia [14], it still causes lymphoproliferative diseases in naturally infected NHPs (i.e. NHP ATL), as it is the case for HTLV-1 in humans [15, 16].

Foamy viruses are also members of the *Retroviridae* family. Even if more than 100 cases of Human Foamy Virus (HFV) infection of zoonotic origin have been reported to date, the number of HFV-infected carriers is unknown (for reviews see [17, 18]). HFV inter-human transmission does not seem to occur and the consequences of this infection *in vivo* remain unknown [17, 19]. Thus, this virus is considered as non-pathogenic in humans, although a recent report demonstrated hematological abnormalities [20]. HFV simian counterpart, SFV, is highly prevalent in NHPs [21]. It has co-evolved with Old World primates for 30 to 100 million years [22], is well adapted to these hosts and is considered non-pathogenic [23]. SFV is readily transmitted among individuals via the saliva, and some reports suggest that in NHPs, SFV active replication is mainly restricted to epithelial cells of the oral mucosa [24]. However, viral DNA is also detected in CD4+ and CD8+ T-cells and in B cells *in vivo* [25], and it was previously suggested that PBMCs represent a site of foamy virus viral latency [26].

To date, no report has demonstrated either *in vitro* or *in vivo* that a given cell could be simultaneously infected with more than one retroviral species. However, cases of natural HTLV-1/HIV-1, HTLV-1/HFV or HIV-1/HFV [27–32] as well as STLV-1/SFV or STLV-1/SIV-1 co-infections [33–35] have been reported. HIV-1/HTLV-1 co-infection has a significant impact on HTLV-1 as well as on HIV-1 pathogenesis in humans [27, 36]. As an example, co-infected patients have a higher risk of HTLV-1-induced neurological disease, inflammatory conditions and leukemia [27, 37]. In addition, a lower HIV-1 viral load was found to be associated with a higher HTLV-1 proviral load (PVL) in humans, while anti-HIV-1 therapy in HIV-1/HTLV-1 co-infected carriers increases HTLV-1 PVL. These results strongly suggest that both retroviruses compete for CD4+ T-cell infection.

Although HIV-1/HFV co-infection has been reported to occur *in vivo* [32], pathogenesis-related data have only been obtained in NHPs. Indeed, co-infected animals have a decreased CD4+ T-cell population and an accelerated death rate [38]. Consistently, persistent infection with foamy virus increases HIV-1 cell-to-cell transmission *in vitro* [39].

These results imply that retroviral co-infection can have a strong effect *in vivo*, even if a given cell might not simultaneously be infected with both retroviruses.

We have access to a colony of baboons (*Papio anubis)* through the CNRS primate center of Rousset-sur-Arc, France. A series of individuals is naturally co-infected with STLV-1 and SFV, while others are only infected with SFV. These animals are followed up on a yearly basis and blood samples are taken routinely. We previously showed that the ratio between CD4+ and CD8+ T-cells remains constant between STLV-1-positive and STLV-1-negative animals and that STLV-1 infection increases the proportion of effector CD8+ T-cells [40]. Given that STLV-1 and SFV may compete for CD4+ T-cells infection *in vivo*, we investigated here, in a series of 36 animals, whether STLV-1 co-infection impacted or not SFV proviral load in the blood and in the saliva. This could then result in an increased risk of zoonotic transmission in case of severe bites [41]. As all animals in the cohort were infected with SFV, we could not decipher whether SFV infection impacts STLV-1 PVL. Of note, STLV-1 provirus has never been searched in saliva, while studies on the presence of HTLV-1 provirus in this fluid, which contains lymphocytes, are rare [42, 43], and there is no evidence that HTLV-1 could be transmitted through this fluid. We first demonstrate that STLV-1 provirus can be found in the saliva in half of the infected animals that were tested, which is not negligible. In addition, STLV-1/SFV co-infection does not affect SFV PVL in the saliva but leads to a very significant increase in SFV proviral load in the blood. In addition, we show that PTLV-1 Tax protein can activate foamy LTR. These results demonstrate that STLV-1 co-infection has some beneficial effect on SFV replication *in vivo*. This may influence the ability of the virus to be zoonotically transmitted.

## Methods

### Ethics statement

The use of animals was approved by ethic committee (APAFIS#4227–201604130940121) from French Minister of education and research. Animals are housed at the primate center of the CNRS (UPS 846) in Rousset-sur-Arc and cared for in compliance with French regulations. All individuals live in social groups and have free access to outdoor areas connected to indoor areas. Enclosures are enriched by wooden platforms and vertical structures. Baboons are fed four times a day with monkey pellets, seed mixture, fresh vegetables and fruits. Water is available ad libitum. The experimental procedure complied with the current French laws and the European directive 86/609/CEE. Blood and saliva samples were obtained after anesthesia. Animals were cared for in compliance with French regulations. Animals were anesthetized by ketamine and Medetomidin to allow blood (at the saphenous vein) and saliva collection.

### Animals

Thirty-six SFV naturally infected baboons (*Papio anubis*) were included in this study. Eighteen animals were STLV-1 uninfected and eighteen were STLV-1 naturally infected as determined by immunofluorescence with HTLV-1 infected MT-2 cells confirmed by western-blot (MP diagnostics HTLV-1/II 2.4 for some of them). All animals but one were females.

### Blood samples

Whole blood was obtained by venipuncture from the internal saphenous vein and transferred into Vacutainer tubes containing EDTA. Peripheral blood mononuclear cells (PBMCs) were separated on Ficoll gradient. Briefly, blood was diluted 2-fold in PBS-2mM EDTA before loading on Ficoll-Paque PLUS (GE Healthcare). After centrifugation (30 min 400g at room temperature), PBMCs were collected, washed in PBS-2mM EDTA and frozen in 90% SVF, 10% DMSO. High-molecular-weight DNA was then extracted from frozen PBMC samples using the NucleoSpin Tissue kit (Macherey Nagel) with slight modifications, as follows. Cells were defrosted in complete medium, counted and aliquoted (5 million by tube). Lysis was performed for 1 hour at 56°C and elution was performed with 90 μl of elution buffer supplied in the kit. DNA concentration was then measured (NanoDrop ND-1000 Spectrophomometer, Thermo Scientific).

### Saliva samples

Saliva and buccal cells were obtained by harvesting 1–2 ml of PBS deposited in the mouth of the animals at the jwol level without scrapping cheeks. After centrifugation for 10 min at 400g, high-molecular-weight DNA was extracted using the NucleoSpin Tissue kit (Macherey Nagel). DNA concentration was measured (NanoDrop ND-1000 Spectrophomometer, Thermo Scientific).

### SFV real-time PCR

Real-time PCR (qPCR) was performed with 20–40 ng of genomic DNA for β-actin amplification and with 100–120 ng of genomic DNA for LTR amplification, using the FastStart Universal SYBR Green Master (Roche), 0,2μM of each primer (PBF3 and 3R see below) using the SYBR Green Master mix (Roche) on a StepOnePlus system (Applied Biosystem) in a total volume of 50μl. After denaturation at 95°C for 10 min, the reaction mixtures containing DNA were cycled 50 times at 95°C for 15 sec, 60°C for 30 sec. The melt curve was then performed as follow: 15 sec at 95°C, 1 min at 60°C followed by a gradual increase until 95°C with acquisition signal every 0.7°C. As a positive control that was used for all experiments, a fragment of *P*. *anubis* SFV 5'LTR was amplified using the same primers i.e. PBF3 (5’ AAAGATATTGTATATTAGCCTTGCT) [44] and 3R (5’ CACGTTGGGCGCCAATTG) [45]. Amplification of human β-actin (Act-F 5′ TGAGCTGCGTGTGGCTCC and Act-R 5′ GGCATGGGGGAGGGCATACC) was done on each experimental sample. A series of diluted amplified products was obtained and used for normalization (i.e. SFV copy numbers for 100.000 cells). Sensitivity of the protocol was determined to be 10 copies/for 100ng of genomic DNA.

### STLV-1 real-time PCR

Real time PCR was performed with 20–40 ng of genomic DNA for β-actin amplification and with 100–120 ng of genomic DNA for *tax* amplification, using the FastStart Universal SYBR Green Master (Roche) containing ROX (Carboxy-X-Rhodamine) as an internal reference, in 50 μl of final volume. Samples were incubated for 10 min at 95°C; then 50 cycles were performed using a StepOnePlus system (Applied Biosystem) (10 sec at 95°C and 30 sec at 60°C), and then the melt curve was performed between 60°C and 95°C. Tax and β-actin primer efficacy was determined on a series of specific amplicon dilutions. Specific Tax primers Tax-F 5′-GTTGTATGAGTGATTGGCGGGGTAA and Tax-R 5′-TGTTTGGAGACTGTGTACAAGGCG were used. Amplification of human β-actin was done as described above and allowed to determine STLV-1 copy numbers for 100.000 cells. Sensitivity of the protocol was determined to be 10 copies/sample.

### Transfection and luciferase assay

10^6^ Jurkat cells were transfected with 100 ng of foamy LTR-luc construct or of HTLV-1 LTR-luc construct in the presence of increasing amount of Tas [46, 47] or of Tax [48] constructs (0-800ng) using the Superfect reagent (Qiagen). Transfections were carried out in the presence of a renilla luciferase vector (phRG-TK) in order to normalize for transfection efficiency. Thirty-six hours after transfection, cells were lysed and luciferase activity was measured using the Dual-luciferase assay system (Promega).

### Statistical analyses

Graphs and statistical analyses were performed with the GraphPad Prism software. Comparison of PVL and of age between the different groups was performed using non-parametric unpaired T test (Mann-Whitney test). Comparison of the PVL in the saliva and in PBMCs was performed using non-parametric paired T test (Wilcoxon test, _***_ = p-value < 0.001; _****_ = p-value < 0.0001 and ns = not significant). Comparison of frequencies was performed by a *χ*^2^ test.

## Results

SFV and HTLV-1 proviruses, *i*.*e*. viral DNA integrated into the host genome, have been found in T-cells and saliva *in vivo* [25, 43]. Free SFV viral particles, *i*.*e*. viral RNA in cell-free samples, can also be found in saliva, while HTLV-1 particles are extremely rare and poorly infectious, as a result of which cell-cell contact is required for an efficient viral transmission [49]. We wanted to assess the effect of STLV-1 co-infection on SFV proviral load (PVL) in saliva and blood, and therefore the putative inter-individual viral transmission through infected cells. We therefore took advantage of a cohort of *Papio anubis*, and analyzed eighteen animals naturally co-infected with SFV and STLV-1, and the same number of animals that were only SFV-positive. The animals from both groups had a similar age (S1 Fig).

**Fig 1:**
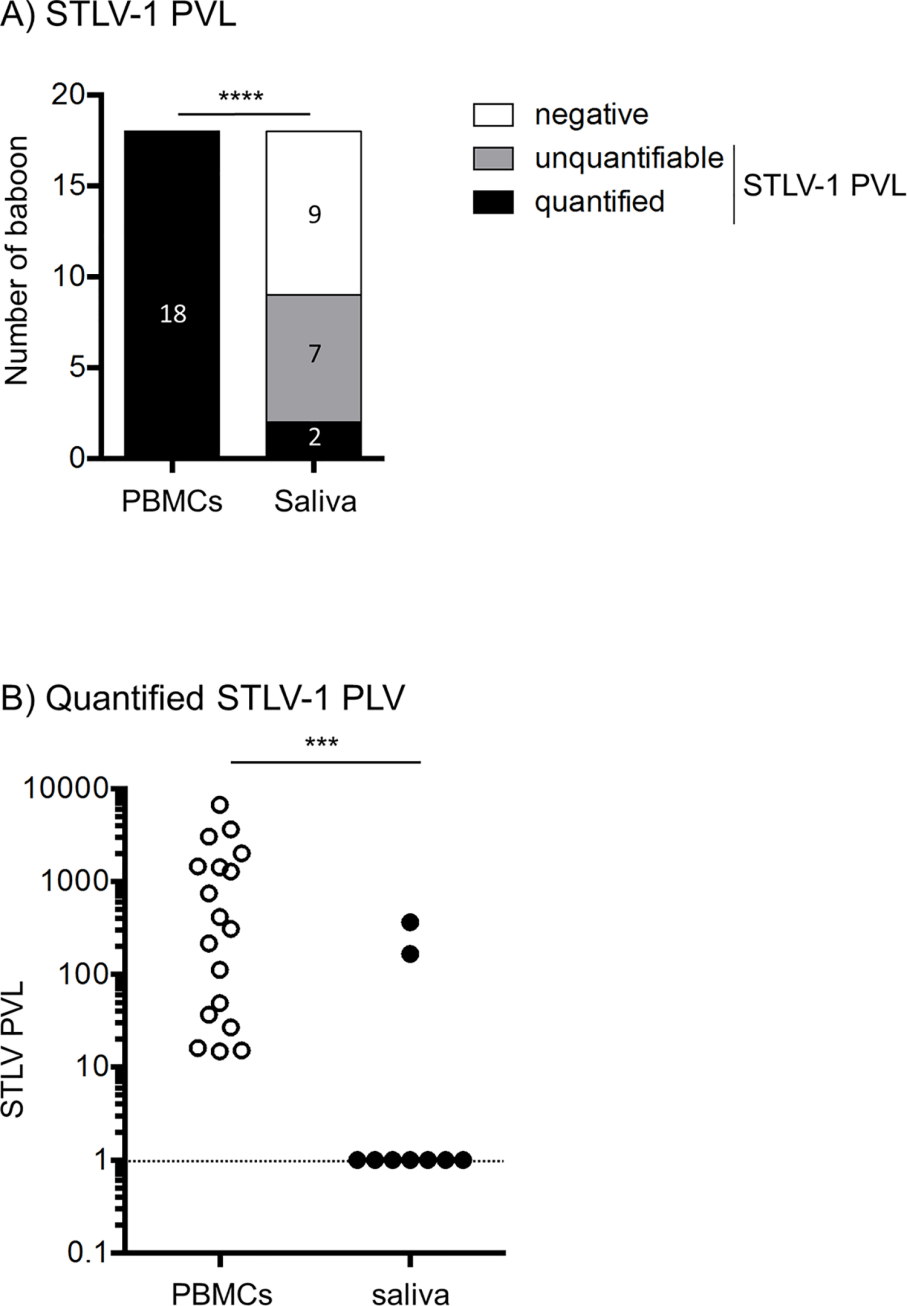
STLV-1 proviral load in PBMCs and saliva. (A) Number of baboons with a quantified STLV-1 PVL (black box), a positive but non-quantifiable STLV-1 PVL (grey box) and a negative STLV-1 PVL (white box). *χ*^2^ test, **** p<0.0001. (B) Quantified STLV-1 PVL in PBMCs (open circles) and saliva (black circles) samples. The dotted line represents the threshold of quantification by real-time PCR. Mann-Whitney test, *** p<0.001.

Because STLV-1 provirus has never been searched in saliva, we first assessed STLV-1 PVL in both the blood and saliva for each STLV-1-infected animal, by qPCR (Fig 1). Samples were classified as negative, positive but non-quantifiable (i.e. a quantifiable signal at least twice among 2 to 3 qPCR experiments, each performed in duplicate) but STLV-1 *tax* or SFV LTR copy number beyond the amplicon standard range in addition to a positive signal visible on a gel loaded with the PCR products, S2 Fig) or positive and quantified (positive qPCR and *tax* copy number within the amplicon standard range). Interestingly, saliva samples analyzed by qPCR were positive for STLV-1 proviral DNA in 9/18 animals, including 7 positive but non-quantifiable and 2 positive and quantified samples (Fig 1A). As a control, qPCR analyses performed on the blood showed positive and quantified results in all animals. The frequencies of negative, positive but non-quantifiable and positive and quantified samples among blood or saliva samples were significantly different (Fig 1A, p<0.0001). We then compared quantified STLV-1 PVL in the blood and in the saliva (Fig 1B). As expected, quantified STLV-1 PVL were significantly higher in the blood compared to the saliva (Fig 1B, median value for PBMCs = 363.7 and median value for saliva = 266.7, p<0.001). There was no significant correlation between the PVL in the blood and in the saliva since a high PVL in the blood was not associated with a high PVL in the saliva. This result suggests that HTLV-1 PVL in the blood cannot be used as a prediction factor for the presence of the virus in cells present in the saliva. Taken together, these results indicate that the STLV-1 provirus can be found in the saliva in half of the infected animals, which is not negligible.

**Fig 2:**
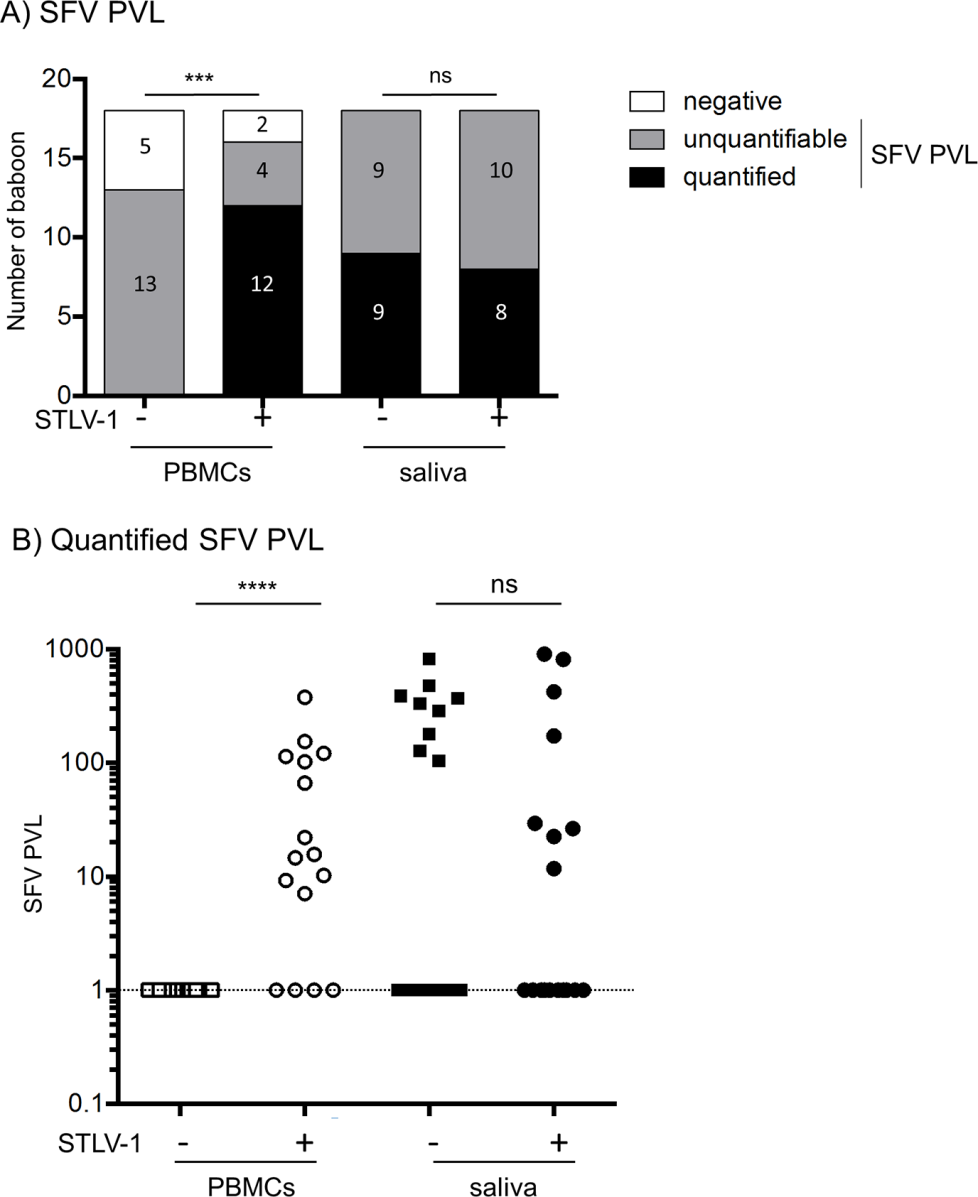
SVF proviral load in PBMCs and saliva. (A) Number of baboons with a quantified SFV PVL (black box), a positive but non-quantifiable SFV PVL (grey box) and a negative SFV PVL (white box). *χ*^2^ test, *** p<0.001, ns: non-significant. (B) Quantified SFV PVL in the saliva. Squares represent SFV mono-infected animals and circles represent STLV-1/SFV co-infected animals. Bars represent median values. The dotted line represents the threshold of quantification by real-time PCR. Mann-Whitney test, **** p<0.001 ns: non-significant.

To decipher whether STLV-1 infection impacts SFV replication, we then performed SFV proviral load analysis by qPCR in the blood and saliva of animals that were or not co-infected with STLV-1 (Fig 2). As for STLV-1, samples were classified as negative, positive but non-quantifiable (positive qPCR but LTR copy number beyond the amplicon standard range) or positive and quantified (positive qPCR and LTR copy number within the amplicon standard range). First, we did not observe any difference in the frequencies of negative, positive but non-quantifiable and positive and quantified samples among STLV-1-negative or STLV-1-positive samples in the saliva (Fig 2A, right columns). The quantified SFV PVL values in the saliva of STLV-1-negative and STLV-1-positive animals were not significantly different (Fig 2B median value = 334.3 in SFV animals and 100.8 in SFV/STLV-1 co-infected animals, saliva values). Of note, there was no correlation between SFV PVL and STLV-1 PVL values in samples obtained from the 12 quantifiable samples (S3 Fig).

Regarding blood samples, even if the frequency of positive samples was similar between STLV-1-negative and positive groups (13 vs. 12+4 animals), the frequencies of negative, positive but non-quantifiable and positive and quantified samples were significantly different (Fig 2A, left columns, p<0.0001). Indeed, there was no animal where the SFV PVL could be quantified among STLV-1-negative animals, while 75% (i.e. 12 out of 18) of samples were quantified among SFV/STLV-1 co-infected animals. Thus, the SFV PVL in the blood is much higher in STLV-1 co-infected animals, indicating that STLV-1 co-infection may impact SFV replication. These results also suggest that exposure to blood from co-infected animals should be linked to a higher risk of SFV transmission.

**Fig 3:**
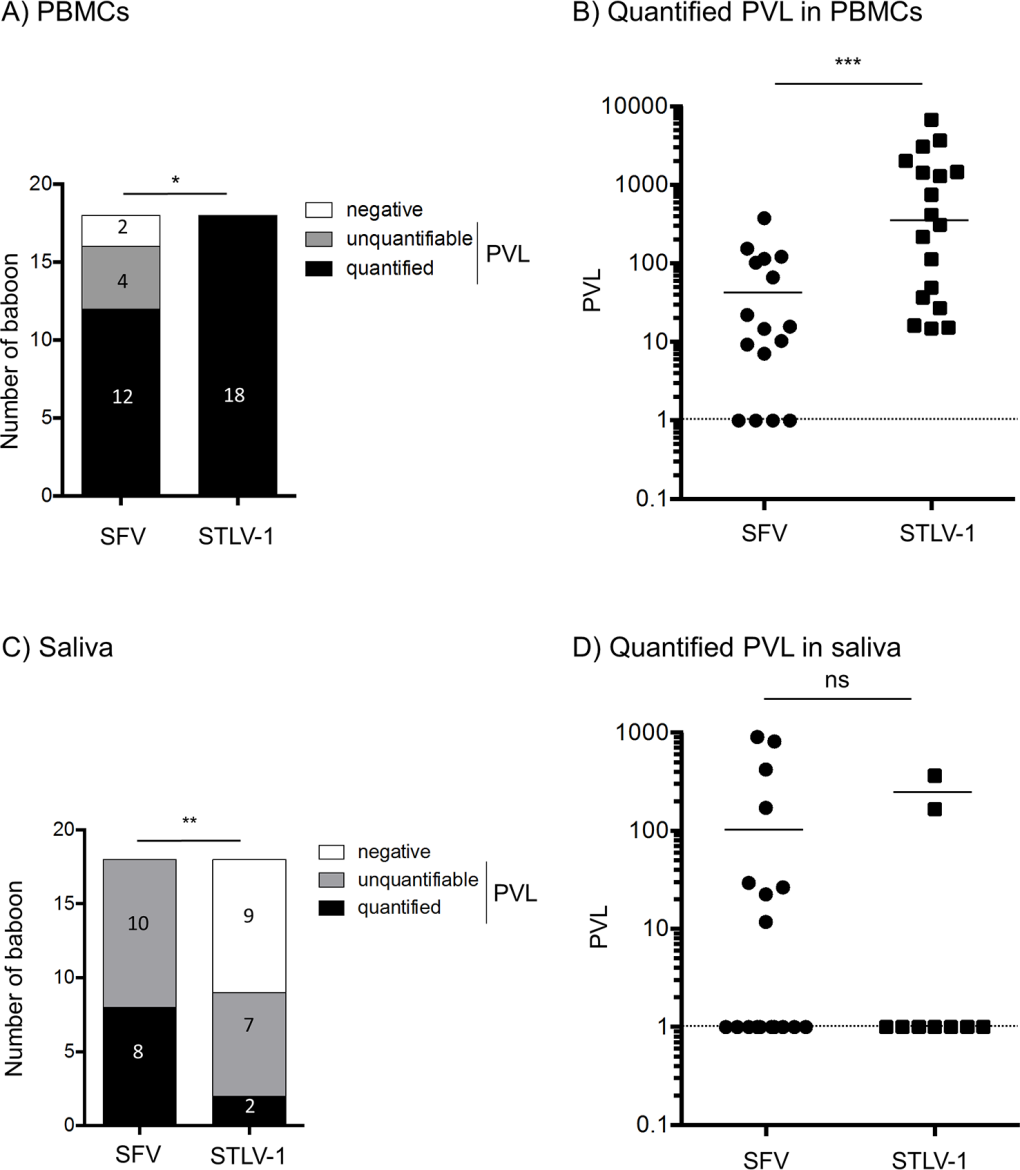
Comparison of STLV-1 *vs*. SVF proviral load in blood and saliva samples. (A and C) Number of infected baboons with a quantified SFV or STLV-1 PVL (black box), a positive but non-quantifiable SFV or STLV-1 PVL (grey box), and a negative SFV or STLV-1 PVL (white box) in the blood (A) and in the saliva (C). *χ*^2^ test, * p<0.05, ** p<0.01. (B and D) Quantified SFV and STLV-1 PVL in PBMCs (B) and in the saliva (D). The dotted line represents the threshold of quantification by real-time PCR. Bars represent median. Wilcoxon test, *** p<0.001, ns: non-significant.

Finally, we compared STLV-1 and SFV PVL in blood and saliva of co-infected animals (Fig 3). The frequencies of negative, positive but unquantifiable and positive and quantified blood samples were significantly different between STLV-1 and SFV (Fig 3A, p = 0.025). STLV-1 PVL were also significantly higher than quantified SFV PVL in PMBCs (Fig 3B, median value for SFV = 50,21 and median value for STLV-1 = 363,7, p<0.001). Similar analyses were performed with saliva samples. The frequencies of distribution in 3 categories (negative, unquantifiable- and quantifiable-positive animals) were significantly different between two viruses (Fig 3C, p = 0,0014). However, contrary to results obtained in the blood, the quantified PVL values were not significantly different between the two viruses (Fig 3D, median value for SFV = 100,82 and median value for STLV-1 = 266).

**Fig 4:**
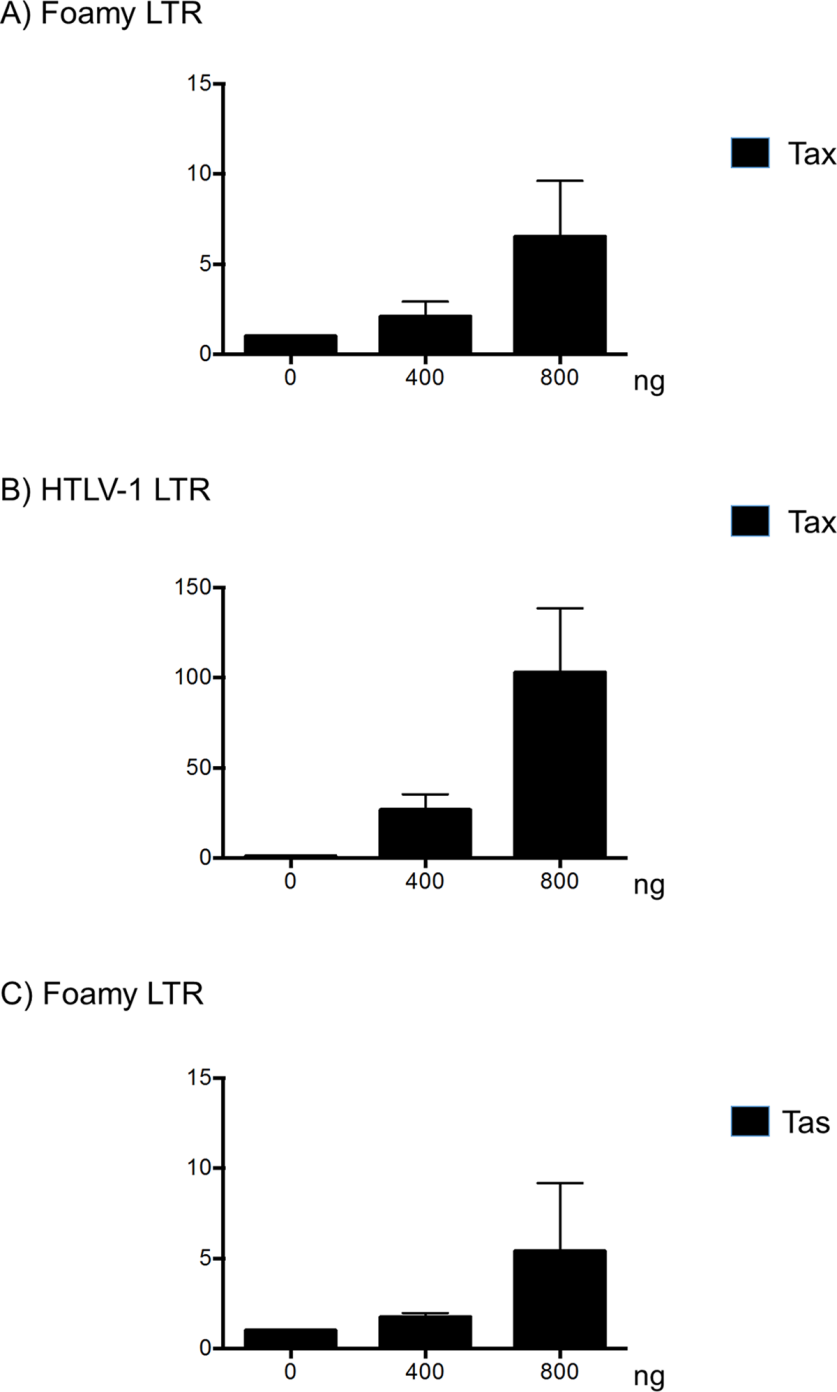
Tax protein can activate foamy promoter. 10^6^ Jurkat cells were transfected with foamy virus LTR-luc construct (A and C) or HTLV-1 LTR-luc (B) together with increasing among of HTLV-1 Tax (A, B) or foamy Tas (C). Thirty-six hours later, luciferase activity was measured and normalized. Results are shown as mean of 2 independent experiments.

Since it was shown that Tax protein is also present under a soluble cell-free form [50, 51], it is therefore possible that such version of the viral protein is present *in vivo* in naturally infected animals where it could enter foamy infected cells. We then tested whether Tax can activate transcription from the foamy promoter in Jurkat T-cells (Fig 4). Indeed, and although less efficient than Tas, Tax can activate transcription from foamy-LTR-luc construct in a dose-dependent manner. This observation might explain the observed increase SFV PVL in co-infected animals.

## Discussion

In this report, we first tried to detect STLV-1 provirus in saliva samples obtained without scratching cheeks. Our results clearly demonstrate that STLV-1 DNA is detected in the saliva in 50% of all infected and asymptomatic animals tested. Of note, STLV-1 PVL in saliva is much lower than in PBMCs. Such a study has not been frequently performed with HTLV-1, and, to our knowledge, never with STLV-1. Some results have suggested that, when proviral HTLV-1 is detected in the saliva as well as in epithelial cells and lymphocytes present in saliva, neutralizing antibodies are also present and prevent HTLV-1 transmission through saliva [42, 43, 52, 53]. However, given the fact that it is now well established that STLV-1 can be transmitted to humans through sever bites [8, 41], it would be interesting to determine if there is a minimal STLV-1 PVL value in NHP saliva required for putative STLV-1 transmission through bite. Unfortunately, while blood samples from people who had been bitten have been obtained, neither the NHP source nor its saliva were available.

Given the high prevalence of SFV among NHPs, humans are bitten by NHP that are probably all SFV-positive, and, in some cases STLV-1-positive. Our results clearly demonstrate that co-infection has a positive impact on SFV proviral load in PBMCs but not in the saliva. Thus, the risk of becoming infected by SFV after being bitten by an SFV/STLV-1 co-infected animal, compared to a SFV-only infected animal, should not be different. However, people exposed to the blood of SFV/STLV-1 co-infected animals during bush meat consumption [54] likely have a higher risk of becoming SFV-infected.

Previous reports have shown that HTLV-1 infected cells produce soluble Tax protein that can act as a viral transactivator on cellular genes [50, 51]. The observation of a positive effect of STLV-1 co-infection on SFV PVL suggested either that cells are truly co-infected with both viruses, or, alternatively, that soluble Tax transactivator enters cells already infected with SFV and acts on its promoter. Our results demonstrate that indeed Tax is able to activate transcription from the foamy LTR. Although this does not exclude the fact that true co-infection exists in animals, it is sufficient to suggest that Tax expression and capture leads to an increase in SFV viral replication and thus an increased SFV PVL. It remains to determine whether soluble Tax is secreted from STLV-1-infected cells *in vivo*, whether it escapes anti-Tax antibodies present in the plasma, and if it can activate SFV LTR in SFV-infected cells. Why this positive impact of STLV-1 co-infection is not visible in the saliva remains to be determined. Foamy infection has not been so far associated with a disease either in non-human primates or in humans. However, it is worth noting that foamy proviral load is usually low in vivo, and this might explain the lack of symptoms. The major difference between SFV proviral load in co-infected animals vs. mono-infected ones suggests that it would be of interest to pay more attention to these animals. As an example, one should perform autopsy and compare results with those of mono-infected animals in order to detect any change.

Of note, we did not have any SFV-negative and STLV-1-positive animal in our simian cohort. This prevented any study aimed at determining whether SFV infection is linked to an increased STLV-1 PVL compared to animals that would be STLV-1-mono-infected, although this would be of interest to gain a better insight into the mutual interactions between both retroviruses. Given that humans co-infected with both viruses or only with HTLV-1 are present in Africa, it would then be interesting to determine whether they are at higher risks for developing an HTLV-1 associated disease compared to HTLV-1 infected individuals. However, performing epidemiological studies will be difficult, due to the fact that these individuals are so far in very limited number.

In conclusion, using *in vivo* samples, our results demonstrate for the first time the presence of STLV-1 and SFV retroviruses in the blood and saliva of infected asymptomatic baboons. It would be of interest to perform similar studies among animals living in the wild, although this will be technically very difficult. Our results also strongly suggest that STLV-1 co-infection as a positive effect on SFV replication in the blood, thus suggesting that STLV-1 co-infection might allow a higher rate of inter-individual and possibly inter-species transmission of foamy virus after exposure to infected blood.

## Supporting information

S1 Fig Similar ages in both group of studied non-human primates. Unpaired t-test was used.
(TIF)

S2 Fig STLV-1 Tax PCR results in PBMCs and saliva samples. 2% agarose gels were loaded with PCR products. 213bp is the size of the Tax PCR-product, 107bp is the size of the actin PCR product.
(TIF)

S3 Fig Lack of correlation between STLV-1 PVL and SFV proviral load. Spearman test was used.
(TIF)
