# Supplementary figures and images for: STLV-1 co-infection is correlated with an increased SFV proviral load in the peripheral blood of SFV/STLV-1 naturally infected non-human primates
Source: PLoS Negl Trop Dis. 2018 Oct 1;12(10):e0006812. doi: 10.1371/journal.pntd.0006812 (PMC6181429; doi:10.1371/journal.pntd.0006812)

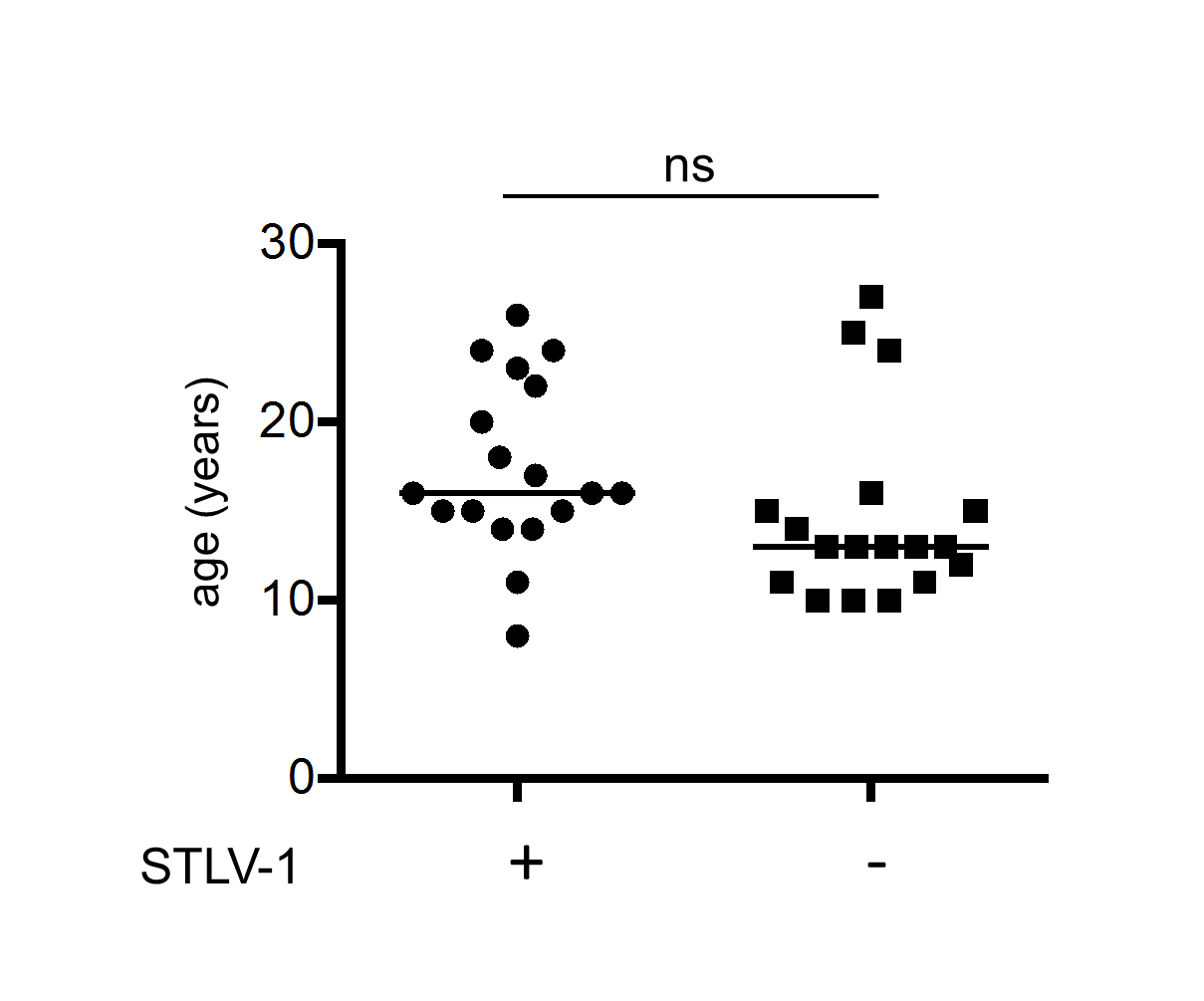

Supplement: S1 Fig — Unpaired t-test was used. (TIF) [file pntd.0006812.s001.TIF]

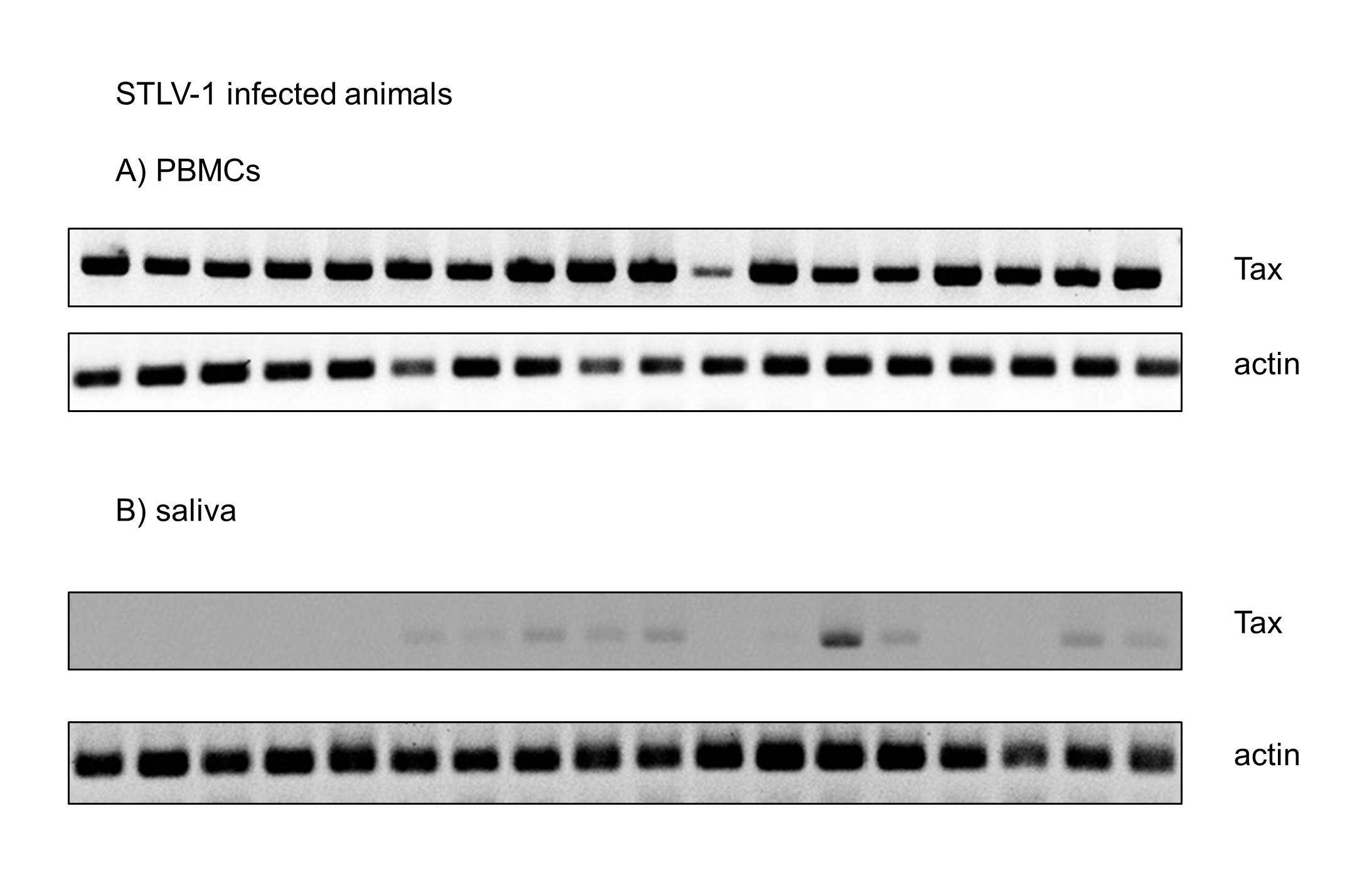

Supplement: S2 Fig — 2% agarose gels were loaded with PCR products. 213bp is the size of the Tax PCR-product, 107bp is the size of the actin PCR product. (TIF) [file pntd.0006812.s002.TIF]

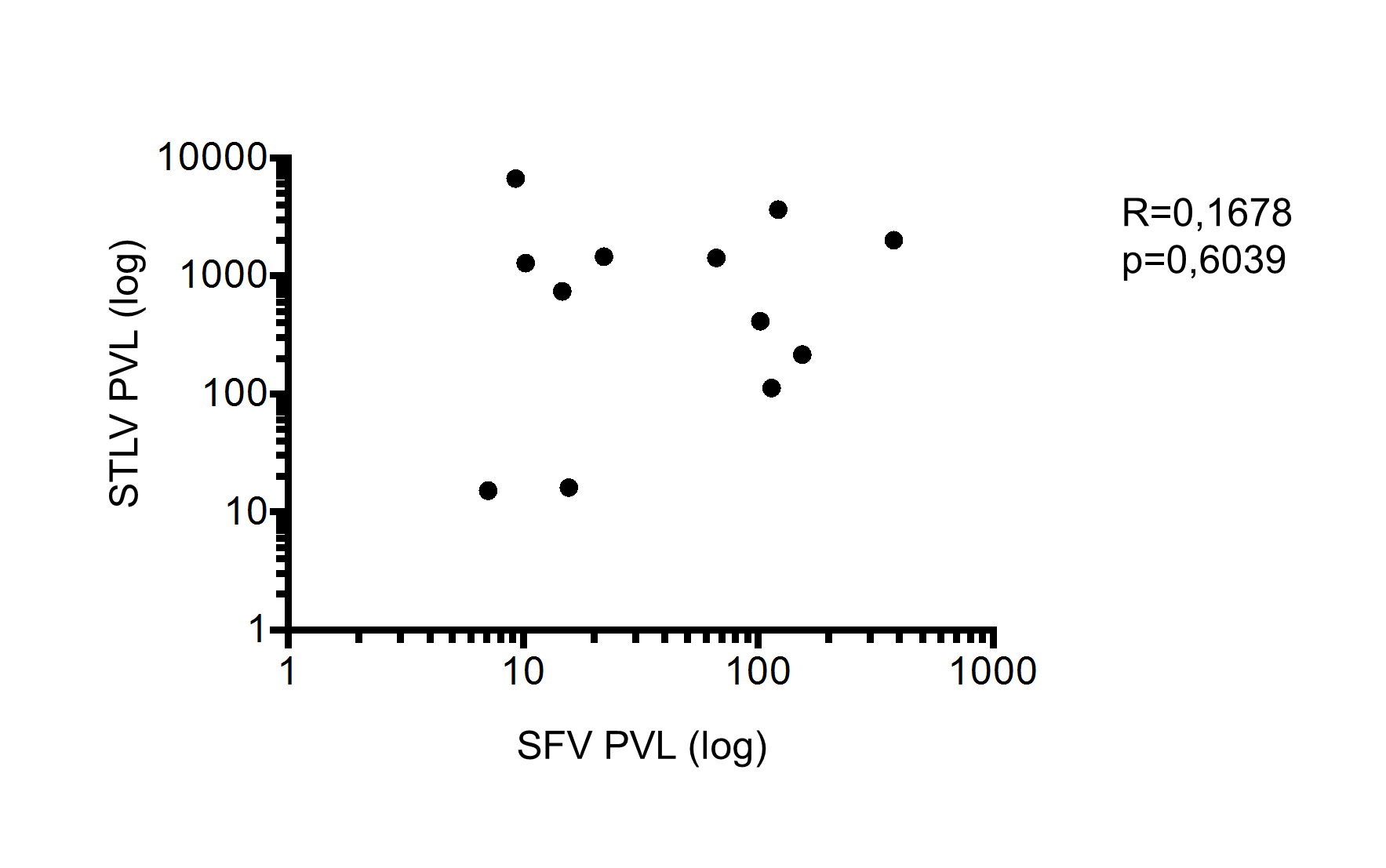

Supplement: S3 Fig — Spearman test was used. (TIF) [file pntd.0006812.s003.TIF]
